# Trimester-specific reference intervals for hemoglobin A1c in non-diabetic pregnancy in a Chinese population

**DOI:** 10.1186/s12884-023-05980-0

**Published:** 2023-09-19

**Authors:** Yuguo Deng, Danling Cheng, Guilian Liao, Xiaoyu Tan, Jinying Yang

**Affiliations:** Department of Obstetrics, Longgang District Maternity & Child Healthcare Hospital of Shenzhen City (Longgang Maternity and Child Institute of Shantou University Medical College), Shenzhen, 518172 Guangdong China

**Keywords:** Glycated hemoglobin, Non-diabetic, Pregnancy, Reference intervals

## Abstract

**Background:**

Physiological glycated hemoglobin (HbA1c) values in each trimester are not well defined. This study aimed to determine trimester-specific reference intervals for HbA1c levels in non-diabetic pregnant women in China.

**Methods:**

In this cross-sectional study, 5,042 Chinese pregnant women from 6 to 41 weeks of gestation were screened. An inclusion of 4,134 non-diabetic women was made to determine the reference intervals, they were divided into three trimesters: trimester 1 (T1), 6 weeks to 13 weeks + 6 days, trimester 2 (T2), 14 weeks to 27 weeks + 6 days, and trimester 3 (T3), 28 weeks to 41 weeks + 6 days. A total of 4,134 women (T1 n = 760, T2 n = 1,953, and T3 n = 1,421) provided blood samples which were analyzed for HbA1c concentrations. HbA1c was measured using high-performance liquid chromatography. The median and percentile (2.5th to 97.5th) for the HbA1c reference intervals were calculated for each trimester.

**Results:**

In total, 8,732 HbA1c measurements were taken. Reference intervals for HbA1c expressed as median and percentile (2.5th to 97.5th) for each trimester were: T1: 4.7 (4.0–5.5%), T2: 4.5 (3.9–5.3%), and T3: 4.8 (4.1–5.7%) respectively. The HbA1c levels were significantly lower in the second trimester compared to those in the first trimester (p < 0.0001), and higher in the third trimester compared to the second trimester (p < 0.0001).

**Conclusions:**

The reference intervals for HbA1c levels were 3.9–5.7% with upper limits of 5.5% in the first trimester, 5.3% in the second trimester, and 5.7% in the third trimester. These findings highlight the importance of considering trimester-specific reference intervals for HbA1c in non-diabetic pregnant women to promote maternal and fetal health.

## **Introduction**

Gestational diabetes mellitus (GDM) is one of the most common pregnancy disorders prone to serious outcomes such as ketoacidosis, preeclampsia, macrosomia, growth restriction, fetal distress, neonatal hypoglycemia, and later cardiovascular disease [1, 2]. The prevalence of GDM varies globally, with rates ranging from 1 to 30% depending on screening methods, diagnostic criteria, geography, and ethnicity [1, 3]. To minimize the negative impact of GDM on pregnancy outcomes, strict glycemic control is crucial. Current antenatal care and management rely on self-managing blood glucose measurements, as recommended by the American Diabetes Association (ADA) and the International Association of the Diabetes and Pregnancy Study Groups (IADPSG), in evaluating glucose control in diabetic pregnant women [4]. However, compliance with blood glucose monitoring is not always consistent, thus alternative methods to assess glycemic control are needed.

HbA1c levels are common clinical indicators for glycemic control in non-pregnant individuals. It reflects an average glycemia for the past 8–12 weeks, can be measured without fasting making it easier for patients and the results are relatively stable and repeatable [5, 6]. However, HbA1c during normal pregnancy is investigated with in one trimester or two trimesters [7, 8], and a few in early pregnancy. Pregnant women with high HbA1c values above normal (5.7 − 6.4%, or around 38 to 47 mmol/mol) are prone to preterm delivery and preeclampsia [8–11]. Early diagnosis of abnormal HbA1c levels allows for preventive measures to be taken.

Studies have established reference intervals for HbA1c levels during pregnancy in non-diabetic women across gestation periods in other ethnic populations [8, 12]. However, there is limited information available on HbA1c levels in early pregnancy in China. A significant association has been found between maternal glucose levels at 10–14 weeks of pregnancy and birth weight at term [13]. Factors such as genetic background and insufficient sample size may contribute to varying HbA1c values in different ethnic populations. Therefore, the HbA1c levels in the early trimester remain undefined and require further research.

The objective of this study was to establish the reference intervals for HbA1c in non-diabetic pregnancy in China. A large, hospital-based sample of pregnant women was recruited. HbA1c in each trimester was measured using a high-performance liquid chromatography (HPLC) assay standardized to the Diabetes Control and Complications Trial (DCCT) values.

## Materials and methods

### Study participants

**Fig. 1:**
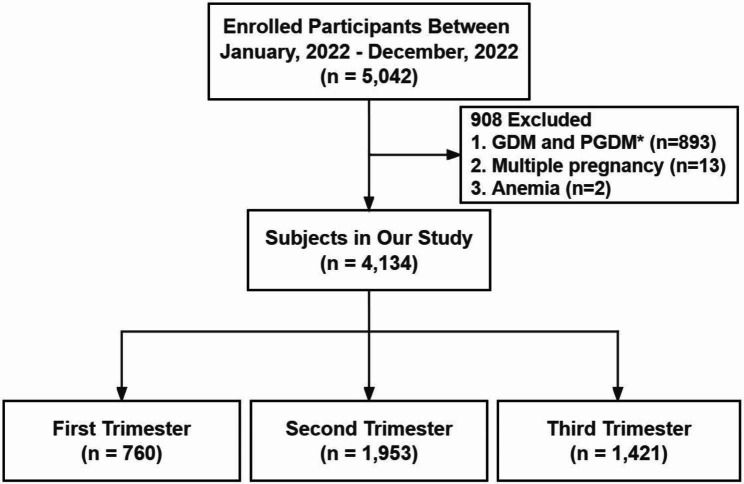
Flowchart illustrating women enrolled in the study. Abbreviations: GDM, gestational diabetes mellitus; PGDM, pre-gestational diabetes mellitus

The baseline data was obtained from the Medical Birth Registry of Shenzhen, China. The sample consisted of 5,042 pregnant women who received regular maternity checkups and delivered at the Longgang District Maternity & Child Healthcare Hospital, one of the largest birthing institutions in Shenzhen City. HbA1c levels, demographic and initial prenatal visit information and medical history were obtained from standard antenatal forms completed by clinical providers during the prenatal screen. Of the 5,042 participants, those with GDM or pregestational diabetes mellitus (PGDM) in their medical records (n = 893), those diagnosed with anemia (n = 2), and multiple pregnancies (n = 13) were excluded. Thus, 4,134 non-diabetic women remained in the study (Fig. 1). During the period from January 1, 2022, to December 31, 2022, a total of 8,732 HbA1c measurements were collected between 6 and 41 weeks of gestation. Demographics and clinical characteristics are demonstrated in Table 1. Participants provided informed consent before the collection of blood samples, and the study was approved by the Institutional Ethics Committee of Longgang District Maternity & Child Healthcare Hospital (No. LGFYYXLL-2022-001).

**Table 1:** Descriptive Frequencies of Demographic and Early Pregnancy Covariates for The Study Participants according to Trimester

Characteristic		First Trimester (n = 760)	Second Trimester (n = 1,953)	Third Trimester (n = 1,421)
		Mean (SD) or % (n)	Mean (SD) or % (n)	Mean (SD) or % (n)
Age, years, Mean (SD)		31.26 ± 4.35	30.78 ± 4.23	30.96 ± 4.37
<35, % (n)		79.5 (604)	82.9 (1619)	82.1 (1166)
≥ 35, % (n)		20.5 (156)	17.1 (334)	17.9 (255)
Gestation weeks, Median [IQR]		9.0 [8.0, 11.0]	24.0 [23.0, 26.0]	34.0 [30.0, 37.0]
Pre-pregnancy BMI, kg/m^2^, % (n)				
	<18.5	15.5 (118)	13.0 (254)	13.2 (187)
	18.5–23.9	63.6 (483)	65.0 (1270)	63.5 (903)
	24.0–27.9	17.2 (131)	18.1 (353)	18.8 (267)
	≥ 28	3.7 (28)	3.9 (76)	4.5 (64)
Height, cm, Mean (SD)		158.66 ± 5.46	158.64 ± 5.33	158.71 ± 5.39
Gravidity, time % (n)				
	1	36.2 (275)	36.6 (714)	35.0 (497)
	2	29.3 (223)	32.6 (636)	32.3 (460)
	≥ 3 times	34.5 (262)	30.9 (603)	32.7 (464)
Parity, time % (n)				
Nulliparous		50.1 (381)	49.1 (959)	46.9 (667)
Multiparous		49.9 (379)	50.9 (994)	53.1 (754)
Hemoglobin (g/L), Mean (SD)		123.8 ± 10.3	115.4 ± 11.6	112.9 ± 11.9

Abbreviations: SD, standard deviation. IQR, interquartile range. BMI, body mass index. BMI was calculated as: BMI = weight (kg) / height ^2^ (m^2^). The subgroups were divided by BMI according to the Chinese BMI guidelines. Data are presented as means ± standard deviation (SD) for continuous variables and frequencies (percentages) for categorical variables

### Obstetric measurements

HbA1c testing was conducted at least once using a standardized laboratory procedure as described by Ho. et al. [14] Blood was collected in the morning after an 8-hour fast and sent to our central laboratory which is equipped with the National Glycohemoglobin Standardization Program (NGSP) certified instruments and the kit for the standardization of HbA1c measurement [15, 16]. The laboratory is participated in the National External Quality Assessment program undertaken by the National Center for Clinical Laboratories (China). HbA1c levels were reported as %. The HbA1c was measured using the HPLC method performed on a Bio-Rad Variant II automated analyzer (Bio-Rad, Hercules, CA); inter-assay coefficient of variations (CVs) < 1%, and the inter-batch CVs < 2% according to the manufacturer’s instructions. Quality control was strictly carried out. The normal range of HbA1c was 4.0–6.0%. A 75 g glucose tolerance test was carried out following overnight fasting. If any of the glucose values met the thresholds as follows: fasting ≥ 5.1 mmol/L (92 mg/dL), 1 h ≥ 10.0 mmol/L (180 mg/dL), 2 h ≥ 8.5 mmol/L (153 mg/dL), GDM was diagnosed according to the criteria of ADA. Participants with PGDM were required to meet the following standards: having a fasting plasma glucose level of ≥ 7.0 mmol/L (126 mg/dL), a 2-h result ≥ 11.1 mmol/L (200 mg/dL) during oral glucose tolerance test, an HbA1c level of ≥ 6.5% or classic symptoms of hyperglycemia with random blood glucose ≥ 11.1 mmol/L (200 mg/dL) [17]. The gestational age and viability were determined by ultrasound. The first trimester (T1) of pregnancy was defined as 6 weeks to 13 weeks + 6 days, the second trimester (T2) as 14–27 weeks and 6 days, and the third trimester (T3) as 28–41 weeks and 6 days. The primary outcome was the reference intervals of HbA1c levels in each trimester.

### Covariates

The following covariates were analyzed: maternal age (in years), height, gravidity (classified as 1, 2, or ≥ 3), parity (distinguished between nulliparous and multiparous), and pre-pregnancy body mass index (BMI). Weight in kilograms divided by square height was used to calculate BMI. Participants were classified into four categories based on the Chinese BMI criteria [18]: underweight (< 18.5 kg/m^2^), normal weight (18.5–23.9 kg/m^2^), overweight (24.0–27.9 kg/m^2^), and obese (≥ 28.0 kg/m^2^). Weight and height were measured while the women wore light clothing and were barefoot according to standard procedure.

### Statistical analysis

Data processing and statistical analysis were performed with SPSS V.22.0 software (IBM, Armonk, NY, United States). Shapiro-Wilk test was used to examine the data normal distribution. Normally distributed data values were expressed as mean (standard deviation), while non-normally distributed parameters were reported as the median (interquartile range). The Kruskal-Wallis test was carried out to compare the medians between groups. Dunn’s post hoc test was then applied to correct for multiple comparisons. The significance threshold was set to P < 0.05.

## Results

### Baseline characteristics

The baseline characteristics of the participants are presented in Table 1. A total of 5,042 pregnancies were recruited during the study period. Out of these, 908 were excluded due to GDM or PGDM (n = 893), multiple pregnancies (n = 13), or anemia (n = 2). Therefore, 4,134 singletons, non-diabetic pregnancy individuals were identified and divided into three groups: T1 (n = 760), T2 (n = 1,953), and T3 (n = 1,421). Pregnant women with non-diabetic pregnancies had 8,732 HbA1c measurements, and diabetic participants had 2,607 determinations.

### The primary outcome

**Fig. 2:**
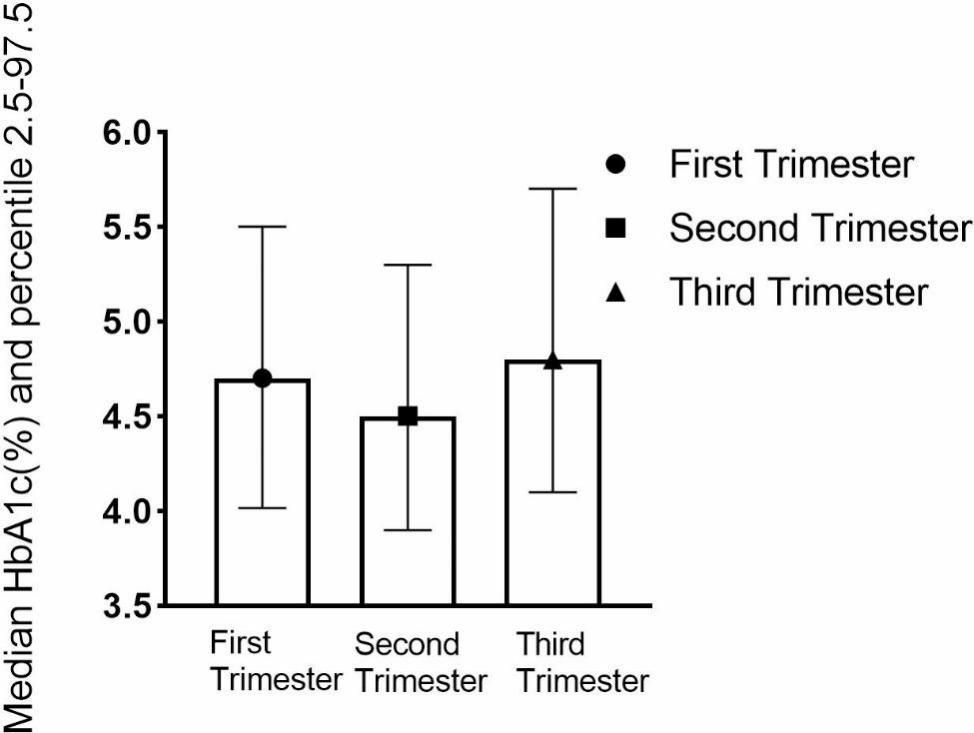
Median and percentile (2.5th to 97.5th) for HbA1c (derived DCCT, %) for non-diabetic pregnancy women for each trimester. The HbA1c values expressed as median and percentile (2.5th to 97.5th) were: T1: 4.7% (4.0–5.5%), T2: 4.50% (3.9–5.3%), and T3: 4.8% (4.1–5.7%), respectively. The T1, T2, T3 were correspond to the first, second and third trimester. To convert HbA1c percent to mmol/mol: IFCC HbA1c unit (mmol/mol) = [10.93×DCCT/NGSP unit (%)] − 23.50. Abbreviations: GDM, gestational diabetes mellitus; PGDM, pre-gestational diabetes mellitus

The HbA1c reference intervals for the T1, T2, and T3 groups are summarized in Fig. 2. The HbA1c values expressed as median and percentile (2.5th to 97.5th) were: T1: 4.7% (4.0–5.5%), T2: 4.50% (3.9–5.3%), and T3: 4.8% (4.1–5.7%), respectively. There was a significant decrease in HbA1c levels from T1 to T2 (p < 0.0001) and an increase from T2 to T3 (p < 0.0001) (Table 2). In addition, the mean HbA1c levels for measurements in all non-diabetic pregnancy women was 4.7 ± 0.4%.

**Table 2:** Dunn’s post-hoc in nondiabetic pregnant women in the study

Comparison of Median HbA1c values	*P values*
Trimester 1 vs. Trimester 2	< 0.0001
Trimester 1 vs. Trimester 3	< 0.0001
Trimester 2 vs. Trimester 3	< 0.0001

Abbreviations: glycated hemoglobin, HbA1c.

The participants had a median gestation week of 9.0, 24.0, and 34.0 for T1, T2, and T3, with interquartile ranges of 8.0–11.0, 23.0–26.0, and 30.0–37.0, respectively. The mean and standard deviation of maternal hemoglobin were T1 (123.8 ± 10.3), T2 (115.4 ± 11.6), and T3 (112.9 ± 11.9) g/L.

## Discussion

The study reported the HbA1c reference intervals (2.5th and 97.5th percentile) in 4, 134 non-diabetic pregnant Chinese women as: 4.0 − 5.5%, 3.9 − 5.3%, and 4.1 − 5.7% in the first, second, and third trimester respectively. Results showed that HbA1c decreased in the second trimester compared to the first trimester, then increased physiologically in the late trimester. We highlighted that preexisting diabetes or GDM treatment goals (for example, HbA1c targets < 6% recommended by ADA [19]) should consider specific times in the gestational period to avoid hypoglycemic episodes in clinical practice. These findings demonstrate the importance of assessing blood glucose fluctuation using trimester-specific HbA1c measurement during pregnancy.

The HbA1c reference intervals in non-diabetic Chinese pregnant women in the 97.5th percentile for the T1, T2, and T3 groups were ≤ 5.5%, 5.3%, and 5.7%, respectively. The results were per previous studies that assessed HbA1c in normal pregnancies. For example, a study of 250 healthy pregnant women in the Netherlands indicated that HbA1c: 4.2–5.4% in early pregnancy, 3.9–5.5% in mid-pregnancy, and 4.1–5.8% in late pregnancy [20]. Also, O’Shea et al. determined that the comparable reference intervals for HbA1c of Irish non-diabetic pregnant women were 4.3–5.4%, 4.4–5.4%, and 4.7–5.7% in the early, middle, and late pregnancy, respectively [21]. A study of 725 normoglycemia pregnant subjects in Mexico showed that the range of HbA1c was 4.5–5.6% in the first trimester, 4.4–5.5% in the second trimester, and 4.5–5.6% in the third trimester [12]. Hence, the 97.5th centiles for HbA1c in our study were similar to previous studies that reported a range of 4.9 to 5.7% in the first trimester [20, 22] and 5.5–5.9% in the third trimester [8, 23]. However, HbA1c levels ranging from 5.4 to 5.7% in the second trimester were lower than in previous observations [21–23]. This may be attributed to potential variabilities in the study populations, such as ethnicity, maternal BMI et al., and sample size differences in the first trimester among those studies. Notably, the sample size of the two studies was 88 [22], and 84 [12], while our study had 706 participants. Furthermore, the mean (SD) of HbA1c was 4.7 (0.4) %, consistent with data from the Hyperglycemia and Adverse Pregnancy Outcome (HAPO) study of more than 20,000 non-diabetic pregnancies that reported maternal mean (SD) values of HbA1c was 4.7 (0.4) % [24]. The HAPO study explored the relationship between glycemia status at 28 weeks to 32 weeks of gestation and perinatal outcomes, providing the strongest data on this topic worldwide.

There are varying outcomes in the HbA1c change pattern throughout pregnancy. Research shows that biphasic changes in HbA1c measurements, initially declined to a nadir level at 24 weeks of gestation, then increase gradually to summit near term [25]. Other studies have concluded that HbA1c levels are unchanged in normal pregnancy [23] or decrease [7] with advancing gestation. However, our study showed that HbA1c levels were lower in the second trimester compared with the first trimester similar to the studies by [20, 22]. The sample size in each trimester in our study is larger, and we use the HbA1c measurement method with the traceability to the DCCT method. As a concurrence, Sánchez-González et al. [12] documented similar conclusions and indicated the increment of HbA1c levels from the second to the third trimester for healthy pregnancies. Moreover, we report a fluctuation of the mean of HbA1c from 4.7% in the early pregnancy to 4.5% in the second pregnancy to 4.8% in the late pregnancy, suggesting a decline of HbA1c in the mid-pregnancy. This is clinically significant when defining the goal for HbA1c in diabetic individuals. During the second trimester, HbA1c levels above the normal range (5.1 − 6.4%, or 32–45 mmol/mol) were associated with an increased risk of adverse pregnancy complications, such as large for gestation age, macrosomia, preterm birth, and preeclampsia [26, 27]. Furthermore, recent research examining continuous glucose monitoring in pregnant women who do not have GDM according to the IADPSG criteria has revealed irregular glycemic fluctuations in these pregnancies as well [28]. These underscore the necessity for more precise diagnostic criteria to effectively identify hyperglycemia during pregnancy. Thus, mid-pregnancy women who exhibit elevated values in oral glucose tolerance tests despite remaining within the normal limits need close observation. Adopting HbA1c reference intervals based on gestational week could improve obstetrics outcomes.

HbA1c concentration should be accepted as an integrated measure of blood glucose levels in both pregnant and non-pregnant diabetic individuals over the past three-month period. Red cell turnover during gestation can impact HbA1c levels regardless of ambient glucose levels. A reduction in HbA1c concentrations before 20 weeks of gestation could be explained by an increase in new red blood cell production and a recline in fasting glucose levels [6]. Moreover, HbA1c integrates maternal glucose levels with fetal development in the previous weeks. Maternal glucose determinations have been linked to birth weight [3], and HbA1c levels found to have a linear relationship with infant size [9]. In addition, maximum fetal growth occurs in the second trimester [13, 29, 30]. These findings suggest that the fetal-placental unit consumes more glucose in the second trimester compared to the increase in maternal glucose production, leading to a decrease in HbA1c levels. Besides, during pregnancy, healthy women experience a 30% increase in basal endogenous glucose production by the end of gestation and a decrease in peripheral tissue insulin sensitivity of about 50% by late gestation [31]. A previous study showed that a decrease in hemoglobin levels corresponds to an increase in HbA1c [32]. We reported mean maternal hemoglobin levels of 123.8 ± 10.3 g/L in the early trimester and 112.9 ± 11.9 g/L in the late trimester, with the HbA1c levels increasing in the late trimester. Unmanaged iron deficiency can result in an HbA1c rise of 0.1–0.2% (1–2 mmol/mol) [6, 33]. These observations may explain the increase in HbA1c values in the third trimester.

There were some strengths in this study. First, our study included the largest sample size in the early trimester. Second, we assessed the reference intervals of HbA1c measurements in a non-diabetic pregnant woman in a Chinese population.

The potential limitations should be mentioned. First, the observational design of the study raised the possibility of residual confounding and selection bias influencing the results. Second, HbA1c measurement did not adequately capture the short-term fluctuations which is also important in the blood glucose management, making continuous glucose monitoring a valuable tool for future research. Third, the interpretation of our study warrants cautious consideration, primarily due to the large sample size employed. It is important to acknowledge that the sheer size of our study cohort enhances the probability of even minor discrepancies attaining statistical significance, irrespective of their genuine clinical significance. Although the different reference intervals of HbA1c levels in three trimesters might be of interest, they should be considered their clinical relevance. To confirm the real-world significance of our findings, an outcome-derived approach is necessary in future research.

## Conclusion

The reference intervals for HbA1c levels in non-diabetic pregnant women in China was 3.9–5.7%. The upper limit of HbA1c levels is 5.5% in the first trimester, 5.3% in the second trimester, and 5.7% in the third trimester among Chinese pregnant women. Further research is required to clarify the association between HbA1c levels in each trimester and pregnancy outcomes. Establishing reference intervals for HbA1c in non-diabetic pregnant women will aid in the early identification of women at higher risk for hyperglycemia-related adverse perinatal outcomes, improving maternal and fetal health.

## Data Availability

The data that support the findings of this study are availability from the corresponding author on reasonable request.

## Abbreviations

HbA1c: glycated hemoglobin
GDM: gestational diabetes mellitus
PGDM: pregestational diabetes mellitus
ADA: American Diabetes Association
IADPSG: International Association of the Diabetes and Pregnancy Study Groups
(HPLC) method: High-performance Liquid Chromatography
DCCT: Diabetes Control and Complications Trial
NGSP: National Glycohemoglobin Standardization Program
(HAPO) study: Hyperglycemia and Adverse Pregnancy Outcome
